# Supplementary material for: Community-based health insurance, healthcare service utilization and associated factors in South Gondar Zone Northwest, Ethiopia, 2021: A comparative cross-sectional study
Source: PLoS One. 2022 Jul 5;17(7):e0270758. doi: 10.1371/journal.pone.0270758 (PMC9255736; doi:10.1371/journal.pone.0270758)
Supplement: S2 File — (DOCX) [file pone.0270758.s002.docx]

# Annex

**English version information sheet**

Questionnaire Identification Number -----------

My name is ________________________. I am working as a data collector in the research Conducted by Fentaw Teshome. We are trying to assess Community based health insurance and healthcare service utilization in, South Gondar Zone. We would like your honest opinion about the questions.

**Introduction**: The information sheet and consent form are prepared for people who are living in South Gondar Zone and whose age is greater than 18 years and household heads.

**Purpose**: I am hopeful that this research will benefit all communities who are users or non-users of community-based health insurance. I will provide research results to the concerned body for intervention.

**Procedure:** To assess Community based health insurance and healthcare service utilization, South Gondar Zone Northwest, Ethiopia you are invited to take part in this project. If you are willing to participate in this project, you need to understand and say, “Yes” on the agreement form. Then after, you will be interviewed by the data collector. All your responses and the results obtained will be kept confidential by using a coding system whereby no one will have access to your response.

**Risk/ Discomfort**: By participating in this research project, you may feel that it has some
discomfort especially on spending time about 30 minutes. We hope you will participate in the study for the sake of the Benefit of the research result. I am sure there is no risk in participating in this research project. You will not be provided any incentive or payment to take part in this project.

**Confidentiality**: The information collected from this research project will be kept confidential and information about you that will be collected by this study will be stored in a file, without your name, but a code number assigned to it. In addition, it will not be revealed to anyone except the principal investigator and will be kept locked with a key. Right to refuse or withdraw: You have full right to refuse from participating in this research. You can choose not to respond to some or all questions if you do not want to give your response. You have also the full right to withdraw from this study at any time you wish, without losing any of your rights. If you have any questions, you can ask at any time.

If you have, additional questions about the study please contact

**Fentaw Teshome - Principal investigator**

**Tel: +251923738761 Email:** [**fentawtesh6@gmail.com**](mailto:fentawtesh6@gmail.com)

**English version questioner**

| **Part I: Socio-demographic characters of mother** | | | | |
| --- | --- | --- | --- | --- |
| **Circle the appropriate response** | | | | |
| S. no. | Question | Response | Skip | Code |
| 101 | Residence | 1. Urban kebele 2. Rural kebele |  |  |
| 102 | Age | ------------------ |  |  |
| 103 | Marital status | 1. Married 2. Single 3. Divorced 4. Widowed |  |  |
| 104 | Religion | 1. Orthodox 2. Muslim 3. Protestant 4. Catholic |  |  |
| 105 | Level of education | 1. Illiterate 2. Read and write 3. Primary (1-8) 4. Secondary (9-12) 5. Collage and above |  |  |
| 106 | Occupation | 1. Housewife 2. Government employee 3. Private employee 4. Merchant 5. Student 6. Others… |  |  |
| 107 | Are there any children with an age less than 5 years? | 1. Yes 2. No |  |  |
| 108 | Are there any elderly with an age greater than 60 years? | 1. Yes 2. No |  |  |
| Healthcare access-related factors and enrolment of study participants in community-based health insurance | | | | |
| 201 | What is your nearest health institution? | 1. Health post 2. Health center 3. Hospital 4. Other specify |  |  |
| 201 | Household wealth index | 1. Poor 2. Medium 3. Rich |  |  |
| 202 | What is your current health status? | 1. Healthy 2. Unhealthy |  |  |
| 203 | What is your first choice for treatment? | 1. Health center 2. Hospital 3. Private health institution 4. Traditional healer 5. Holly water |  |  |
| 204 | What is your current health status? | 1. Healthy 2. Not healthy |  |  |
| 205 | First choice of place for treatment during illness | 1. Health institution 2. Traditional healer |  |  |
| 206 | Do you have a history of chronic illness? | 1. Yes 2. No |  |  |
|  | Healthcare utilization and community health insurance enrolment | | | |
| 207 | Is there any family member who visits a health institution within 3 months? | 1. Yes 2. No |  |  |
| 208 | If yes, how many times do they visit the health institutions? | _____________________ |  |  |
| 209 | Community health insurance enrolment | 1. Enrolled 2. Non-enrolled |  |  |

| **S. N** | **Wealth index Questions** | **Response** | | | **Coding** | | **Skip** |
| --- | --- | --- | --- | --- | --- | --- | --- |
| 301 | Does any member of this household has owned any agricultural land? | Yes  2. No | | | 1  2 | |  |
|  |  | No | | | 2 | |  |
| 302 | How much (local units) of agricultural land do members of this household own? | (in Hectare) | | |  | |  |
| 303 | Does this household own any livestock, herds, other farm animals, or poultry? | Yes  2. No | | | 1 | | 305 |
|  |  | No | | | 2 | |  |
| 304 | How many of the following animals, does this household own?  **[**probe and mark that all apply, multiple answers are possible] |  | Animals | No. | |  |  |
|  |  |  | Milk cows, oxen  or bulls |  | |  |  |
|  |  |  | Horses, donkey  or mules |  | |  |  |
|  |  |  | Goat |  | |  |  |
|  |  |  | Sheep |  | |  |  |
|  |  |  | Chicken |  | |  |  |
|  |  |  | Beehives |  | |  |  |
| 305 | Which of the following does your household have?  *(Record observation, Multiple responses is possible)* | 1. Electricity  2. Watch  3. Radio  4. Television  5. Mobile Telephone  6. Non-Mobile Telephone  7. Chair  8. Table  9. Bed  10. Electric Mitad  11. Other (specify) | | |  | |  |
| 306 | What is the main current source of drinking water for  members of your household? | 1. Piped water  2. Public Tap/Stand Pipe  3. Borehole  4. Protected well  5. Unprotected well  6. Protected Spring  7. Unprotected Spring  8.River/Ponds/Stream/Dam  9. Other (Specify) _ | | |  | |  |
| 307 | What kind of toilet facility do members of your household usually use?  *(Record Observation)* | 1. Flush to the piped sewer system  2. Flush to septic tank  3. Pit latrine with slab  4. Pit latrine without a slab  5. Ventilated improved pit latrine  6. No facility/bush/field  7. Other (specify) | | |  | |  |
| 308 | What are the main materials of the floor of the house?  *(Record observation)* | 1. Earth/Sand  2. Wood planks  3. Palm/Bamboo  4. Ceramic Tiles  5. Cement  6. Other (specify) | | |  | |  |
| 309 | What are the main materials of the roof of the house?  *(Record observation)* | 1. Thatch/straw  2.Leaf/Earth/ Mud/Cow dung  3.Wood planks, cardboard  4. Finished roof (iron, tin, finished wood, cement, ceramic)  5. Other(specify) | | |  | |  |
| 310 | What is the main material of the exterior walls of the house?  *(Record observation)* | 1. Simple wall with mud or local materials  2. Bamboo or stone with mud, plywood, cardboard  3. Finished walls; cement, brick, stone with cement, wood planks  4. No outside walls  5. Others (Specify) _ | | |  | |  |
| 311 | Which means of transport (vehicles) does any member of your household own? | 1. Bicycle  2. Motor cycle  3. Animal-drawn cart  4. Car/truck  5. Bajaj  6. None | | |  | |  |
