# Supplementary material for: Community-based health insurance, healthcare service utilization and associated factors in South Gondar Zone Northwest, Ethiopia, 2021: A comparative cross-sectional study
Source: PLoS One. 2022 Jul 5;17(7):e0270758. doi: 10.1371/journal.pone.0270758 (PMC9255736; doi:10.1371/journal.pone.0270758)
Supplement: Attachment — Submitted filename: Respons to Reviwers.docx [file pone.0270758.s003.docx]

**Date: 11^th^ May 2022**

**Dear Editor-in-chief**

**RE: MANUSCRIPT NUMBER: PONE-D-22-04561**

Thank you for your prompt feedback on our manuscript. We especially appreciate the valuable critique provided by the reviewers. We have appropriately addressed the requested revisions as indicated below in which the text written in green shows the response. As instructed, we have electronically resubmitted a revised manuscript incorporating various revisions as outlined below. We remain available to further edit/revise the manuscript as you may require. In the meantime, we trust that these revisions meet with your approval.

Yours sincerely,

Fentaw Teshome

(Corresponding author)

**Response to Reviewer**

Journal Requirements:

When submitting your revision, we need you to address these additional requirements.

1. Please ensure that your manuscript meets PLOS ONE's style requirements, including those for file naming. The PLOS ONE style templates can be found at

<https://journals.plos.org/plosone/s/file?id=wjVg/PLOSOne_formatting_sample_main_body.pdf>

and <https://journals.plos.org/plosone/s/file?id=ba62/PLOSOne_formatting_sample_title_authors_affiliations.pdf>

- Thank you for the comment. It was corrected as per the comment.

2. Please include your tables as part of your main manuscript and remove the individual files. Please note that supplementary tables (should remain/ be uploaded) as separate "supporting information" files.

- Thank you for the comment. It was corrected as per the comment.

3. We note that you have stated that you will provide repository information for your data at acceptance. Should your manuscript be accepted for publication, we will hold it until you provide the relevant accession numbers or DOIs necessary to access your data. If you wish to make changes to your Data Availability statement, please describe these changes in your cover letter and we will update your Data Availability statement to reflect the information you provide.

- Thank you for the comment. The Data Availability statement was “The data is available from the corresponding author and will be provided upon a reasonable request.” It was corrected as per the comment.

4. Your ethics statement should only appear in the Methods section of your manuscript. If your ethics statement is written in any section besides the Methods, please delete it from any other section.

- Thank you for the comment. It was corrected as per the comment.

5. Please review your reference list to ensure that it is complete and correct. If you have cited papers that have been retracted, please include the rationale for doing so in the manuscript text, or remove these references and replace them with relevant current references. Any changes to the reference list should be mentioned in the rebuttal letter that accompanies your revised manuscript. If you need to cite a retracted article, indicate the article’s retracted status in the References list and also include a citation and full reference for the retraction notice.

- Thank you for the comment. It was corrected as per the comment.

Additional Editor Comments:

Universal Health Coverage's key component is enhanced utilisations amongst the eligible participants. The manuscript submitted that study participants predominantly belong to the poor (47.6%). Additionally, ~ half of the sampled participants could not read and write. The observations are probably self-explanatory to utilisations and major focus areas to be worked upon by the authorities.

- Thank you for the comment.

The authors could add more to the understanding of the content readers if they could divulge the details about various provisions under CBHI, particularly in the introduction section of the manuscript. Further research into Out of Pocket Expenditures amongst the users of CBHI would further highlight the determinants of utilisations or otherwise.

- Thank you for the comment. The aim of this research was majorly to compare and identify factors of health care utilization among CBHI enrolled and non-enrolled households (usually Out of Pocket Expenditures).

Review Comments to the Author

Reviewer #1: Community-based health insurance is an alternative to bad access to healthcare due to financial constraints. This piece of evidence will aid decision-makers towards strengthening health care utilization through insurance. The manuscript has been well-framed, and the study's robustness is quite reflective. The result section requires minor edits (line no. 236-237).

- Thank you for your constructive comment. It was corrected as per the reviewer's comment.

Reviewer #2: The authors are requested to kindly describe the findings in results and discussion section in past tense preferably (For example lines 236-7).

- Thank you, we appreciate the comment. It was corrected as per the reviewer's comment.

In line 147, the authors are requested to specify the need of specifically mentioning about the elderly population in the inclusion criteria as the same is not reflected in results and discussion.

- The need of mentioning the elderly population in the inclusion criteria was that the elderly population tends to utilize healthcare since they are prone to age-related diseases. This was clearly stated in the result and discussion part of the manuscript. The reviewer can re-visit it in the revised manuscript.

In line 159, the authors are requested to kindly cite reference for "principal component analysis" as the same has been used to categorize the study participants into rich, medium and poor based on the wealth index.

- Thank you, we appreciate the comment. It was corrected as per the reviewer's comment.

In the sampling procedure mentioned at line 174, it is not clear that in each household how many members were interviewed. If only one member was interviewed then what was the selection criteria for selecting that household member? If all the members present at the time of interview in a single household, then what was the selection criteria to select a respondent? Could the authors have used KISH method for household survey? In short, how did they eliminate selection bias, and information bias?

- One member of the household who was the household head was interviewed. We thought that the household head represents the household members and represents the target population. The household head is the one who had better information about CBHI membership and health care utilization. Due to this reason, we didn’t use the KISH method for this study. Since our study was a comparative cross-sectional study, it may not be prone to selection bias and information bias, unlike follow-up studies.

Please explain Table 4 in detail. Does the term "enrolled" in line 242 mean users and non users as mentioned in the table 4? The description is not clear with respect to table. It would be better to explain that out of those study participants who had enrolled themselves for the CBHI, how many were using the services and how many were not? And whether, the difference in the utilization of health services was significant or not?

- Thank you, we appreciate the comment. It was corrected as per the reviewer's comment.

In the discussion section, it is may be advisable to use only proportions rather than using absolute numbers (eg line 253)

- Thank you, we appreciate the comment. It was corrected as per the reviewer's comment.

The statement mentioned in lines 267-8 regarding respondents with chronic illness and their utilization of health services (compared to what?)

- Thank you for the question. It was compared with respondents who had no chronic illness and it was corrected as per the reviewer's comment.
